# Recent Advances of Intraocular Lens Materials and Surface Modification in Cataract Surgery

**DOI:** 10.3389/fbioe.2022.913383

**Published:** 2022-06-08

**Authors:** Chenqi Luo, Hanle Wang, Xinyi Chen, Jingjie Xu, Houfa Yin, Ke Yao

**Affiliations:** Eye Center of the Second Affiliated Hospital, Medical College of Zhejiang University, Hangzhou, China

**Keywords:** biomaterials, intraocular lens, surface modification, biocompatibility, posterior capsular opacification

## Abstract

Advances in cataract surgery have increased the demand for intraocular lens (IOL) materials. At present, the progress of IOL materials mainly contains further improving biocompatibility, providing better visual quality and adjustable ability, reducing surgical incision, as well as dealing with complications such as posterior capsular opacification (PCO) and ophthalmitis. The purpose of this review is to describe the research progress of relevant IOL materials classified according to different clinical purposes. The innovation of IOL materials is often based on the common IOL materials on the market, such as silicon and acrylate. Special properties and functions are obtained by adding extra polymers or surface modification. Most of these studies have not yet been commercialized, which requires a large number of clinical trials. But they provide valuable thoughts for the optimization of the IOL function.

## Introduction

Biomaterials are used extensively in medicine nowadays, such as artificial organs, regenerative medicine, wound repair and bioimaging fields (Karayilan et al., 2021). Ophthalmology is also an important application area of biomaterials (Han H. et al., 2020; Wang K. et al., 2021). They are widely involved in retinal regeneration (Abedin Zadeh et al., 2019), vitreous replacement (Wang T. et al., 2021), contact lenses (Xiao et al., 2018; Ma et al., 2022), and intraocular lenses (IOL) (Čanović et al., 2019). The lens is an important transparent, biconvex-shaped structure for optical functions in the eye. It adjusts the refractive power by changing the diopter so that objects at different distances are imaged on the retina (Fisher, 1977; Hejtmancik and Shiels, 2015). Protein denaturation reduces the transparency of the lens and is caused by the increase of age or the influence of other factors such as trauma, genetics, and metabolic diseases (Bloemendal et al., 2004). This pathological condition is called a cataract. It is the leading cause of blindness in elderly patients (Chen et al., 2021). The cataract surgery removes the opacity lens and replaces it with an artificial, transparent, disc-shaped IOL with certain refractive power. About 15.2 million patients can be cured by replacing IOL through cataract surgery every year (Steinmetz et al., 2021).

IOL are used to substitute the preliminary lens. Since its first application in 1949, IOL had experienced more than 70 years of development. In the beginning, people tried to find materials that were safe, stable and had desirable optical properties. Gradually, with the proposing of specific and personalized demands, materials and techniques are continuously updating (Dick and Gerste, 2021). Advances in IOL include materials and structural designs. In the design of the optical structure, the multiple focal points were accomplished through refraction, diffraction, or two combined surface structures to obtain clear imaging at multiple distances (Alio et al., 2017b); Extended Depth of Focus (EDOF) IOL (Rampat and Gatinel, 2021), toric IOL (Kessel et al., 2016), and Aspheric IOL (Schuster et al., 2013) are designed to improve visual quality. In terms of the non-optical part, the square-edge IOL have been shown to effectively inhibit cell proliferation and reduce postoperative adverse reactions (Maddula et al., 2011). Meanwhile, there are many advances in materials. IOL have gone through a transition from rigid materials to foldable soft materials, and commercially available IOL nowadays are mainly composed of different types of acrylic (Werner, 2021). There will be an overview of them in the next section. Materials are improved to reduce IOL size, prevent adverse effects after IOL implantation and surgery (Topete et al., 2021b), improve biocompatibility (Werner, 2008), provide accommodation ability (Alio et al., 2017a), and filter specific light (Downes, 2016). It is hoped that in the future, the IOL will be able to more closely resemble the original lens, in particular, obtain continuous refractive power modulation. Moreover, IOL with additional therapeutic capabilities are also expected (Toffoletto et al., 2020). There is currently no IOL that fits all needs. Thus, in different conditions, various materials are being studied to improve the IOL on the market.

**FIGURE 1:**
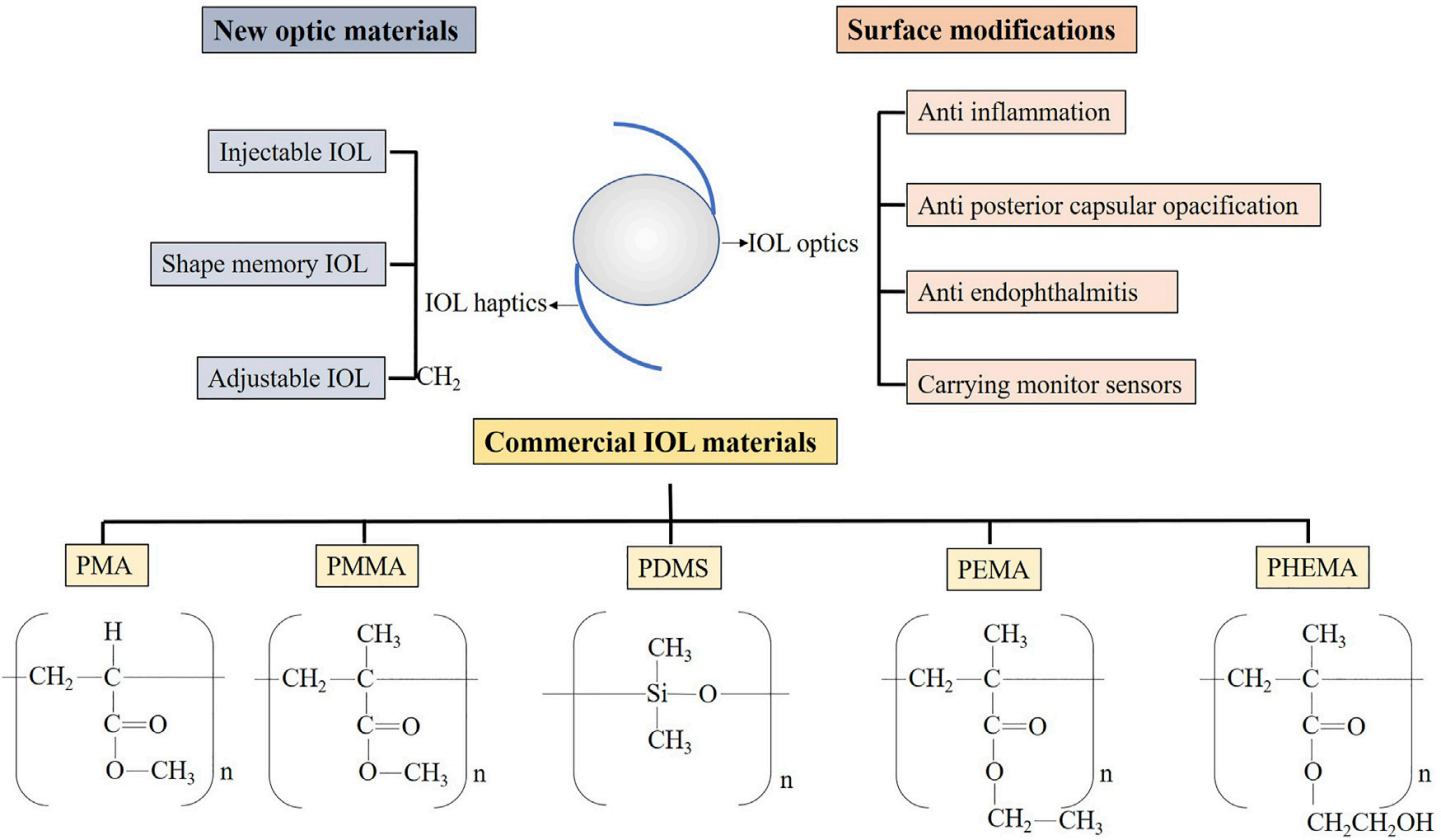
The main outline of this review. PMA, polymethylacrylate; PMMA, polymethylmethacrylate; PDMS, polydimethylsiloxane; PEMA, polyethyl methacrylate; PHEMA, poly2-hydroxyethylmethacrylate.

There were many reviews of IOL published over the years. They usually focused on innovations in structural design, in which case there are more commercialized IOL (Čanović et al., 2019; Dick and Gerste, 2021; Werner, 2021). There were also some reviews on the application of polymer materials in ophthalmology (Kirchhof et al., 2015; Karayilan et al., 2021). They introduced the applications of polymeric materials not only in IOL but also in other ophthalmic fields, such as contact lenses and vitreous replacement. The innovations of IOL with a specific function were other aspects (Huang et al., 2016; Pepose et al., 2017; Toffoletto et al., 2020; Topete et al., 2021b). They tend to focus on advances in IOL for carrying drugs or for therapeutic use and for surface modification to improve biocompatibility. The above types of reviews all discussed the progress of IOL materials, but they often covered only a specific part. In this review, we introduced the material development of IOL from the progress in the aspects of the main optic material and the surface modification. The advances were categorized for different design purposes (Figure 1). In considering that some of them had a very long research history and related reviews had been published already, we mainly discussed studies carried out in the last ten years.

## Materials on the Market for the Manufacture of Intraocular Lenses

The materials in ophthalmic applications should meet the basic requirements, including lasting optical transparency, high resolution, chemical stability, high histocompatibility, and be sterilizable (Allarakhia et al., 1987). The properties of common IOL materials on the market are summarized in Table 1. Polymethylmethacrylate (PMMA) was first introduced in 1949 (Ridley, 1952) as the chemical material to replace the preliminary lens by Ridley. He noticed some injured pilots with plexiglass shards in their eyes. These substances remained in the eye tissue for long periods without much adverse impact (Ridley, 1976). Although PMMA was very hard, difficult to change in shape, and cannot withstand high temperature and pressure, it has been used as an intraocular lens (IOL) without a suitable replacement for decades (Apple et al., 1984). Due to the lack of flexibility, a large incision (5–7 mm) was required to implant the PMMA IOL (Čanović et al., 2019). The advances of phacoemulsification cataract surgery enabling smaller surgical incisions had spurred research into soft, foldable IOL.

**TABLE 1:** Properties of common IOL materials.

Materials	Foldable/unfoldable	Biocompatibility	Advantages	Disadvantages
PMMA	Unfoldable (rigid)	Low rate of inflammatory cell accumulation	Low cost	5–6 mm incision to insert
		High rate of PCO*	Low aqueous flare	
			High visual quality	
PDMS	Foldable (soft)	Low rate of inflammatory cell accumulation	Small incision	Tissue injury during expanding in capsular
		Fibrotic reaction of lens surface		Opacification of surface after contacting with intravitreal air
Hydrophilic acrylic	Foldable (soft)	Low rate of inflammatory cell accumulation	Low aqueous flare	Limitation in shape design
		High rate of PCO calcification	Easy to handle	Long-term IOL opacification
Hydrophobic acrylic	Foldable (soft)	Low rate of PCO	Good visual quality	High aqueous flare
		High rate of inflammatory cell accumulation	Compatible with a sharp-edged design	Tacky surface
				High RI*

*PCO, posterior capsular opacification; RI, refractive index.

The very first foldable IOL used *in vivo* was in 1976 (Allarakhia et al., 1987). In the following period, soft IOL had been extensively studied and applied. One of the soft IOL is made of polydimethylsiloxane (PDMS). Silicone materials are in various structures with a refractive index of 1.41–1.46, higher than that of PMMA (Kapoor and Gupta, 2020). It means that under the same refractive power, the IOL of the silicone material are thinner than PMMA (Čanović et al., 2019). Grafting carbazole on the side chain of polysiloxane has been proved to increase the refractive index of the silicone hydrogel material (Xu et al., 2015). Silicon has been reported to have relatively low biocompatibility and easily adhere to silicone oil and particles when contacting with intravitreal air (Ozyol et al., 2017). This negatively affects the transparency of IOL forwards. In addition, the silicone IOL unfolds very fast after insertion into the eye, and it is likely to injure the capsular bag (Čanović et al., 2019).

The most widely used IOL materials nowadays are acrylic (Olson et al., 2003). They are polymer or copolymer formed by methyl acrylate, methyl methacrylate (MMA), ethyl methacrylate (EMA), and 2-hydroxyethyl methacrylate (HEMA). In the polymerization process, carbon-carbon double bonds open to link molecules and form various structures (Tetz and Jorgensen, 2015). They are less elastic than silicone and take about 3–5 s to fully unfold, making them safer during surgery. Acrylic IOL are mainly divided into hydrophilic and hydrophobic. The water content of hydrophobic IOL is generally 0.5%–1%, while that of hydrophilic IOL is usually 18%–38% (Werner, 2021). The difference is mainly contributed to the content of HEMA in the copolymer, and it is temperature related (Miyata and Yaguchi, 2004).

Hydrophilic IOL can be adapted to smaller surgical incisions (less than 2 mm) by dehydration, thus ideal for microincision cataract surgery (Kohnen and Klaproth, 2010). They are in good compatibility with biological tissues due to high water content (Ozyol et al., 2017) and are reported to be less prone to aggregation of inflammatory cells on the surface (Abela-Formanek et al., 2011). However, studies have found that hydrophilic IOL has a high probability of posterior capsule opacification (PCO) as the hydrophilic surface makes it easier for lens epithelial cells to grow (Ozyol et al., 2017). And calcification has been reported likely to occur in hypertonic hydrophilic surface which allows intraocular metabolites to enter (Werner et al., 2006). In addition, the high-water content can also limit the shape design of the IOL.

Hydrophobic acrylic IOL are more widely used on the market nowadays (Werner, 2021). They can be made thinner with a high refractive index (1.44–1.55) (Čanović et al., 2019). The tacky surface of hydrophobic IOL allows the lens to adhere tightly to the capsule, reducing the proliferation of lens epithelial cells (Katayama et al., 2007), hence decreasing the PCO. Furthermore, a sharp-edged design is more feasible to implement on hydrophobic IOL, which prevent migration of lens epithelial cells to the IOL (Nibourg et al., 2015). The main drawback of hydrophobic IOL is glare caused by a high refractive index (Čanović et al., 2019). Glistening was also found to be more likely to occur in hydrophobic IOL (Tetz et al., 2009). It is because that water that collects in hydrophobic polymers forms pockets that grow as temperature changes. Therefore, properly increasing the water content of the material can be a solution to this problem (Tetz and Jorgensen, 2015).

## Advances in Intraocular Lens Optical Materials

### Material Inserted Through Smaller Incisions

Up to now, phacoemulsification requires an incision within 2 mm (Li et al., 2018). Smaller surgical incisions bring faster postoperative vision recovery, fewer complications, and less postoperative astigmatism (Rao et al., 2018). This puts forward requirements for the development of IOL. The IOL needs to be implanted in the eye through a small incision, with a 6.0 mm optic portion diameter is sufficient size (Werner, 2021). Furthermore, the accommodative function might remain if the lens capsular bag was preserved. Commonly used IOL reduce the size by folding Seward (1997), but some new materials are also developed through shape memory or injection of liquid IOL, which may further reduce the IOL size.

#### Injectable Intraocular Lens

The fluid has no fixed shape, so it can enter the capsular bag through a small surgical incision. The ideal injectable IOL is in a liquid state during the implantation procedure and subsequently forms a stable solid or remains in liquid form. Liquid materials for injectable IOL need to meet the basic conditions: good biocompatibility, non-toxicity, ability to maintain a transparent state, and sufficient refractive power (Karayilan et al., 2021). The process of turning liquid materials into solids *in vivo* may involve some external stimulation, which is preferably non-invasive. In addition, due to the fluidity of the liquid, the filling material may flow out of the incision. Thus, a suitable method of filling the incision is required. Kessler was the first to propose the use of liquid to replace the lens in 1960 (Kessler, 1964). He mainly introduced the surgical method of injecting liquid materials such as silicon fluid and oil immersion through a small incision and observed that the silicon and catalyst were solidified after 30 min *in vivo*.

In the following decades, liquid materials and hardening methods have been tried, mainly focused on silicone and hydrogels (de Groot et al., 2001; Koopmans et al., 2003). Hardening methods include light, heat, enzymatic reaction, etc., Meanwhile, inappropriate external stimuli can also cause indeterminate damage to eye tissue. A polysiloxane macromonomer was used by Hughes Hao et al. (2010) to make injectable IOL. Under the attendance of Karstedt’s catalyst and blue light (70 mW/cm^2^) and a photoinitiator, it would turn into a gel state after about 5 min with a refractive index of 1.47 and good light transmittance. Subsequently, they introduced phenyl to make an adjustable refractive index. But its biological toxicity was uncertain, and the transparency was reported to decrease over time (Hao et al., 2012).

Niu et al. (2010) synthesized the thiol-ene photopolymerization by ultraviolet irradiation. The compounds of three polymers: diacrylate, poly (ethylene glycol) (PEG), and pentaerythritol tetrakis (3-mercapto propionate), were hardened under the radiation of ultraviolet light (4.5 mW/cm^2^) with the presence of a photoinitiator. It was proved to reduce the amount of ultraviolet light required when photopolymerizing, compared to the previous studies (Han et al., 2003), thus decreasing the damage of ultraviolet rays to the eyes. Nonetheless, heat release was observed during the polymerization reaction, resulting in a significant local temperature rise, posing a risk of thermal injury to the eye. Moreover, unreacted polymers might be toxic to cells.

In addition to photopolymer materials, heat-sensitive materials were also developing. In 2012 (Annaka et al., 2012), Annaka reported A modified PEG with silica nanoparticles that solidifies into a gel at physiological body temperature. The injection needed to be done at 45°C when it appeared to be in the form of liquid. It had ideal transparency and refractive index, while the biocompatibility was unclear. Its limitation lies in the temperature requirements at the time of surgical injection. Furthermore, enzymatic crosslink reaction was used to solidify hydrogels by 4-PPO/PEO block copolymer [4-arm poly (propylene oxide)-poly (ethylene oxide)] later in 2014 (Lee et al., 2014). The catalysts were horseradish peroxidase (HRP) and hydrogen peroxide (H_2_O_2_). The gel had young’s modulus similar to that of the initial lens, and no tissue damage was found during the injection and coagulation procedure. More importantly, it was observed with high biocompatibility, especially that the PCO rate was significantly lower than other materials.

**FIGURE 2:**
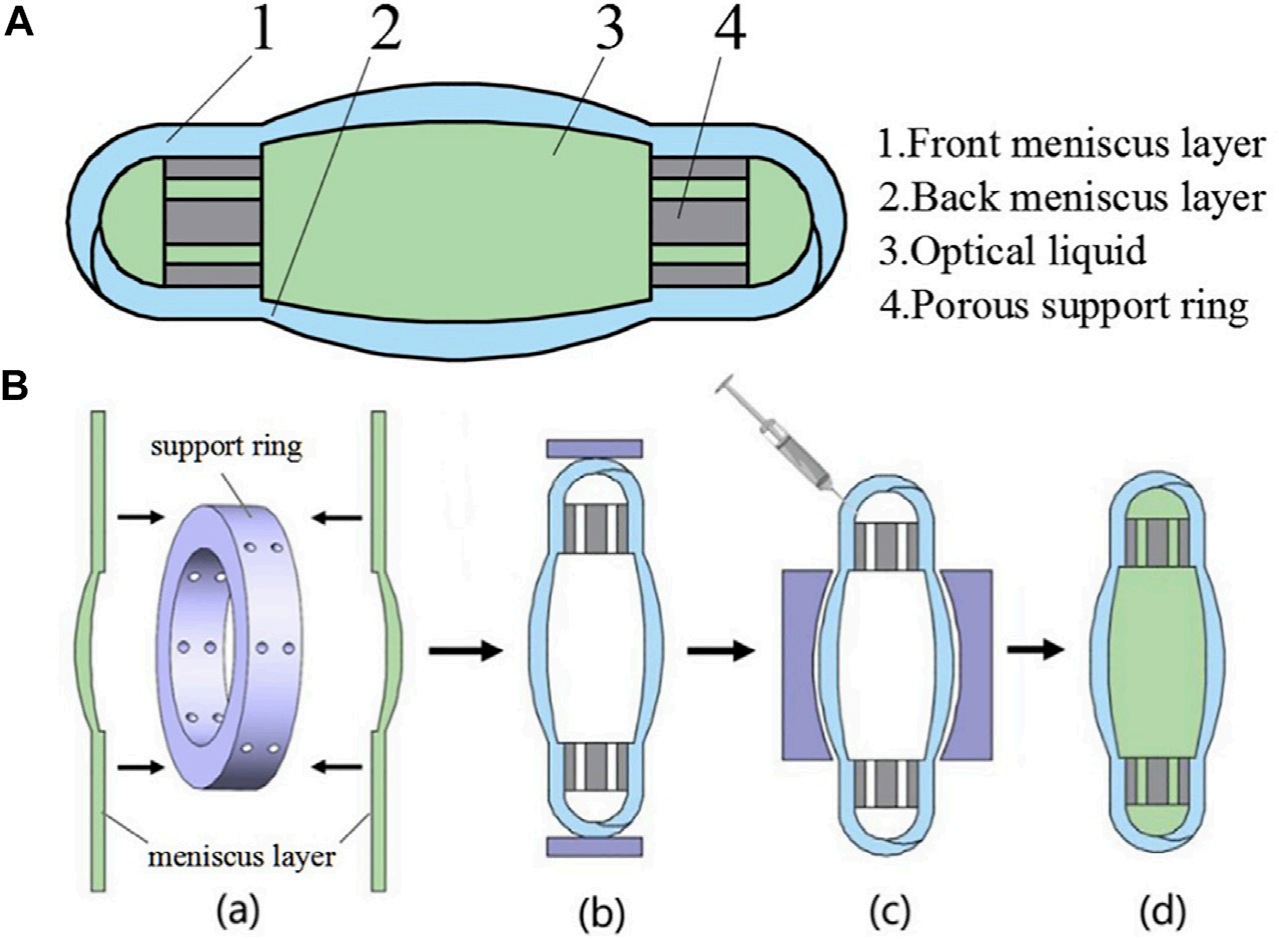
**(A)** The structure diagram of IOL. **(B)** The assembling process of the designed IOL from Liang et al. (2019), with the permission of Elsevier.

Except for coagulating the copolymer in the eye, some studies had attempted to keep it in the capsular bag as a liquid. In this way, the liquid IOL could obtain the ability to alter the shape, thus processing the effect of adjusting the refractive power, which was also called accommodation IOL. The relative research in this area is brought by Deboer et al. (2015). They introduced a deformable PDMS lens shell filled with a transparent liquid. Experiments had found that the liquid in this device did not leak out and maintained good light transmittance. The elastic modulus of the IOL shell was close to that of the native lens, so it could achieve the effect of adjusting the refractive power. Other studies on the combination of elastic membrane and optical fluids designed to manufacture accommodation IOL have also been proposed later (Liang et al., 2015; Liang et al., 2019) (Figure 2).

In conclusion, research progress in injectable intraocular lenses has been relatively slow. Curing methods currently used mainly include light, heat, and catalysts, all of which can cause eye damage to a certain extent. While injecting fluids with maintaining devices will be safer methods. Further exploration is needed to ensure the safety of the materials and inserting process.

#### Shape Memory Intraocular Lens

External stimuli (light, temperature, PH, etc.) cause some polymers to change in shape. When the shape change is irreversible, they are called shape memory polymers (SMPs), and the phenomenon is known as the shape memory effect (SME) (Sun et al., 2019). The use of SMPs material allows the IOL to be pre-deformed to a very small size, requiring only a small surgical incision to pass through. After inserting in the eye, when the environmental conditions change, the IOL will revert to memory. Song et al. (2011) introduced a type of methacrylate SMPs that could be applied in IOL. Ethylene glycol phenyl ether acrylate (EGPEA), ethylene glycol phenyl ether methacrylate (EGPEMA), and long alkyl chain formed the basic structure of SMP. The solid SMP was scrimped to a small size *in vitro* by heating and expanded to a full-size IOL when it reached the body temperature and remained stable. The experiment found that the transition temperature is positively correlated with the concentration of methacrylate. The refractive index of the copolymer was also changed by the concentration of EGPEA and EGPEMA and was reported 1.514–1.499. And the cytocompatibility was proved to be good.

Recently, a series of poly (dimethyl acrylamide-co-stearyl acrylate and/or lauryl acrylate) (PDMAAm-co-SA and/or LA) was synthesized by 3D printing technology (Shiblee et al., 2018). 3D printing technology, rather than traditional mold production, provides the possibility for the customization of personalized IOL. The PDMAAm-co-SA and/or LA thermal set material was proved to have a refractive index of 1.41–1.47, comparable to traditional IOL materials. However, its young’s modulus varies widely and could be high for IOL, and biocompatibility remained unclear.

### Adjustable Intraocular Lens Materials

Failure to achieve target diopter after IOL replacement is a very common problem after cataract surgery for patients (Chee et al., 2015). A recent European survey showed that more than 27% of patients had postoperative refractive power, not within ±0.5 diopter (D) of the target (Lundstrom et al., 2018). In the past, postoperative ametropia often required a second invasive surgery, such as keratorefractive surgery, for correction (Ford et al., 2014). Adjustable IOL were hence designed to avoid secondary surgery.

**FIGURE 3:**
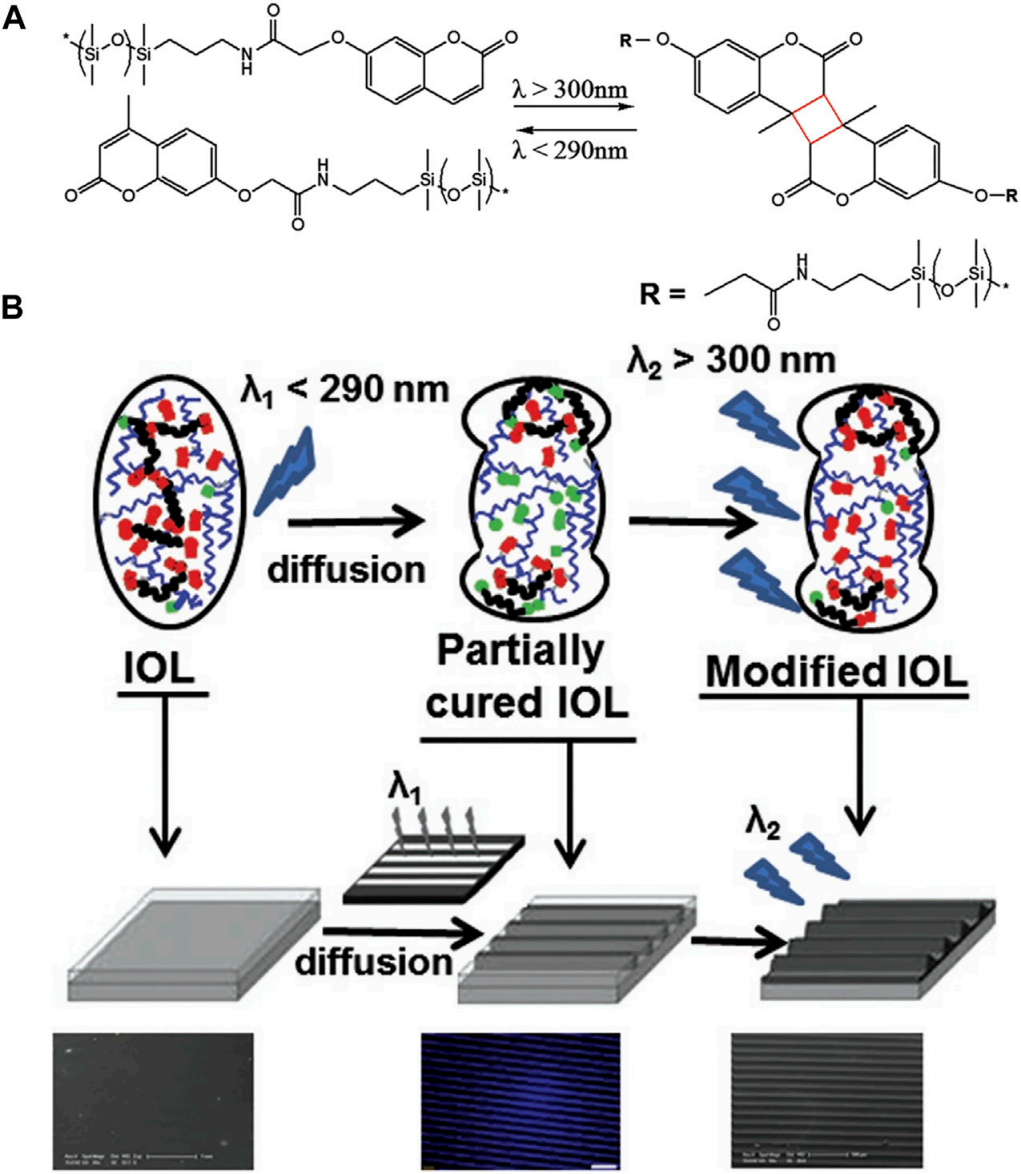
**(A)** Schematic of photoreversible crosslinking of PDSM-coumarin polymer. **(B)** Schematic of designed IOL from Jellali et al. (2017), with the permission of Wiley.

A light-adjustable lens (LAL), produced by Calhoun Vision, Inc., has been officially approved for commercial use for years (Chang, 2019). This LAL was synthesized based on silicon and mainly contained ultraviolet absorbers, photoinitiators, and photosensitive macromolecules. When exposed to ultraviolet light, macromolecules were promoted to polymerize by photoinitiators Dick and Gerste (2021). The macromer that did not participate in the polymerization subsequently diffused into the treated or exposed areas, leading to a change in the shape of the LAL, which ultimately resulted in an alteration in refractive power. Clinical trials had proved that this LAL had the ideal ability to correct visual acuity after cataract surgery, including patients with long or short axial lengths and patients undergoing corneal refractive surgery. It was reported to have modulation capabilities up to 2D (Hengerer et al., 2011; Villegas et al., 2014). However, since the process of adjusting refractive power required the eye to be exposed to ultraviolet light, its negative effects on the retina are concerned. An update to LAL was reported that ultraviolet-absorbing groups were added to reduce eye exposure (Chang, 2019). However, light exposure depleted the number of monomers in the material. Hence the polymerization of macromonomers in LAL was irreversible. Once the diopter of LAL was fixed, it could not be changed anymore.

(Kim et al., 2003; Trager et al., 2008), Schraub and Hampp (2011) proposed a silicon-coumarin polymer-based IOL design that can repeatedly adjust the diopter. Modulation of diopter was accomplished by photoreaction of coumarin side groups, the dimerization of coumarin to form cyclobutane led to a decrease in refractive power *via* crosslinking. On the contrary, its rupture allowed the refractive power to increase. It was proven to achieve a 2.5D adjustment range. With optimization, this process could be achieved by visible light two-photon absorption or ultraviolet single-photon absorption. This represents better protection for the eyes. On this basis, Jellali et al. (2017) synthesized a silicone-coumarin phototunable intraocular lens and tested the photosensitivity and biocompatibility. Ultraviolet-visible light spectroscopy showed that irradiation above 300 nm occurred crosslinking, and the process was reversed under light below 245 nm. It was able to be implanted in the eye in a crosslinked state and did not require special protection of the patient’s eyes from the light (Figure 3).

A thermoplastic material, soft polysiloxane-urea-elastomers (PSUs), was synthesized by Riehle for accommodation IOL Riehle et al. (2018). Amino-propyl-terminated polydimethylsiloxanes, 4,4′-methylenebis (cyclohexylisocyanate), and 1,3-Bis (3-aminopropyl)-1,1,3,3-tetramethyldisiloxane were involved in the synthesis of this material. The material was verified to withstand a mechanical stretch of 5% (1 mm), equivalent to an 8D refractive index change. The transparency and refractive index are desirable for IOL, and it has been proved to have no apparent cytotoxicity. The elastic material avoids light injury to the eyes, but how to stretch the material through the ciliary muscle still needs further research.

Femtosecond lasers are now a widely used assistive technology for cataract surgery. Research has found that femtosecond lasers may affect the properties of polymers. Ding et al. (2006) induced a 0.06 refractive index change in the polymer of the hydrogel under femtosecond laser treatment. This phenomenon was called “laser-induced refractive index change (LIRIC).” Changes in the refractive index of IOL materials were thought to be caused by thermal or alternation in hydrophilicity (Bille et al., 2017). Scientists tried to apply it to produce new tunable IOL, possibly shortly.

In addition, overall material optimization was also designed to improve biocompatibility, which will be introduced in detail in the next section. Based on the previous research (Kannan et al., 2005), Wang et al. (2014) proposed a method to synthesize poly (hedral oligomeric silsesquioxane-co-methyl methacrylate) (allyl POSS-PMMA) polymer and applied it as an IOL material. It had a regular structure, forming a better surface morphology. And it was more hydrophobic than PMMA material. But more animal experiments need to be carried out to verify its biocompatibility.

## Intraocular Lens Surface Modification Materials

### Materials to Improve Biocompatibility

Biocompatibility refers to the ability of a material to fuse with the surrounding tissue. Amon described biocompatibility of the IOL as uveal and capsular biocompatibility Amon (2001). The implantation of allogeneic IOL materials will promote the leakage of proteins and macrophages in the vascular of the uvea. They may adhere to the surface of the IOL and promote inflammatory response (Titiyal et al., 2020). The extent of the inflammatory response induced by IOL is indicative of uveal compatibility. As for capsular biocompatibility, it often manifests as anterior capsular opacity (ACO), PCO, and capsular bag contraction (Trivedi et al., 2002). They are mainly related to the growth of LECs. In addition, the residual LECs on the capsular bag may grow to the surface of the IOL and lead to calcification (Grzybowski et al., 2021).

Improving biocompatibility helps reduce adverse effects after cataract surgery, such as inflammatory reactions, PCO, ACO, etc., And optimizing IOL materials is considered a promising approach to improve IOL biocompatibility. In addition to altering the IOL material, surface modification is also seen as an effective method. It includes layer-by-layer (LBL) self-assembly, plasma surface modification, surface coating, surface grafted, photochemical immobilization, etc., (Huang et al., 2016) Moreover, drugs or chemical molecules are modified on the surface of the material to reduce negative reactions (Topete et al., 2021b).

The polymer with good light transmittance is considered preferentially, and the step of surface modification should also minimize the change to the surface structure of IOL. These reduce the impact on the optical properties of the IOL. In addition, the coating thickness, refractive index, transmittance, swelling capacity and wettability before and after modification also need to be proved to be close by experiments. Different modifications affect the hydrophilicity or hydrophobicity of the IOL surface and this may be the same or opposite to the IOL bulk material. Since hydrophilic and hydrophobic IOL surfaces have their own advantages and disadvantages, the choice needs to be based on actual needs. Some modifications occurred in the non-optical region of the IOL surface, so that its change to the optical properties of the IOL did not affect the IOL function.

#### Materials to Reduce Inflammation

As previously introduced, the improvement of uveal biocompatibility is mainly manifested in the reduction of postoperative inflammation. Research in this area has been ongoing for decades, and heparin-surface-modified (HSM) IOL have been proven reliable and applied in clinical use. At present, the main research direction in this field is grafting hydrophilic or anti-adhesion groups to the IOL surface. The articles involved are listed in Table 2.

**TABLE 2:** The studies on materials reducing inflammation.

Surface modification	IOL optic materials	Technique	Article
HEP*	PMMA		Larsson et al. (1989)
	PMMA, Silicon		Arthur et al. (2001)
	Hydrophobic acrylic		Krall et al. (2014)
	PMMA		Rønbeck and Kugelberg, (2014)
PEG*	Hydrophobic acrylic	APGD^*^	Lin et al. (2010)
NVP*	Hydrophobic acrylic		Wang et al. (2013)
MPC*	Hydrophobic acrylic	Ultraviolt irradiation	Huang et al. (2017)
	Silicon	SI-RAFT*	Han et al. (2017)
MPC-MAA*	Hydrophobic acrylic	Plasma	Tan et al. (2017)
Recombinant hirudin anticoagulant	Hydrophobic acrylic	Ammonia plasma	Zheng et al. (2016)

*HEP, heparin; PEG, poly (Ethylene Glycol); NVP, N-vinyl pyrrolidone; MPC, 2-methacryloyloxyethyl phosphorylcholine; MAA, methyl acrylic acid; APGD, atmospheric pressure glow discharge; SI-RAFT, surface-initiated reversible addition-fragmentation chain transfer.

HSM IOL had been designed for decades and successfully commercialized. It was first synthesized with the initiation of Ce^4+^ ions (Boffa et al., 1979). Heparin and PMMA were polymerized by oxidation reaction. HSM was gradually applied to the surface of various IOL materials, such as PMMA (Larsson et al., 1989) and silicon (Arthur et al., 2001). Heparin provides higher hydrophilicity to the surface of the IOL. It had been proved by experiments those materials with high hydrophilicity cause less postoperative inflammatory reactions due to reduced adhesion of surface cells and proteins (Roesel et al., 2008). Therefore, HSM was thought to improve the uveal biocompatibility of the material, which had also been demonstrated in many clinical trials, especially for patients with preoperative uveitis (Krall et al., 2014; Zhang et al., 2017). Unfortunately, HSM has not shown a special effect on the prevention of PCO (Ronbeck and Kugelberg, 2014) through long-term observation.

Plasma surface modification was applied to change the surface of the silicon IOL by adding 2-methacryloyloxyethyl phosphorylcholine (MPC) (Wang and Liu, 2011). Not only did MPC-IOL exhibit a repulsive effect on proteins, but it had also been shown to reduce the adhesion of silicone oil, which was an important defect of silicon IOL (Huang X.-D. et al., 2013). Similarly, MPCs were grafted onto hydrophobic acrylate IOL together with methyl acrylic acid (MAA) by air plasma treatment in 2017 (Tan et al., 2017). This MPC-MMA IOL had been reported to significantly reduce protein absorption and inflammatory responses than previous MPC IOL.

**FIGURE 4:**
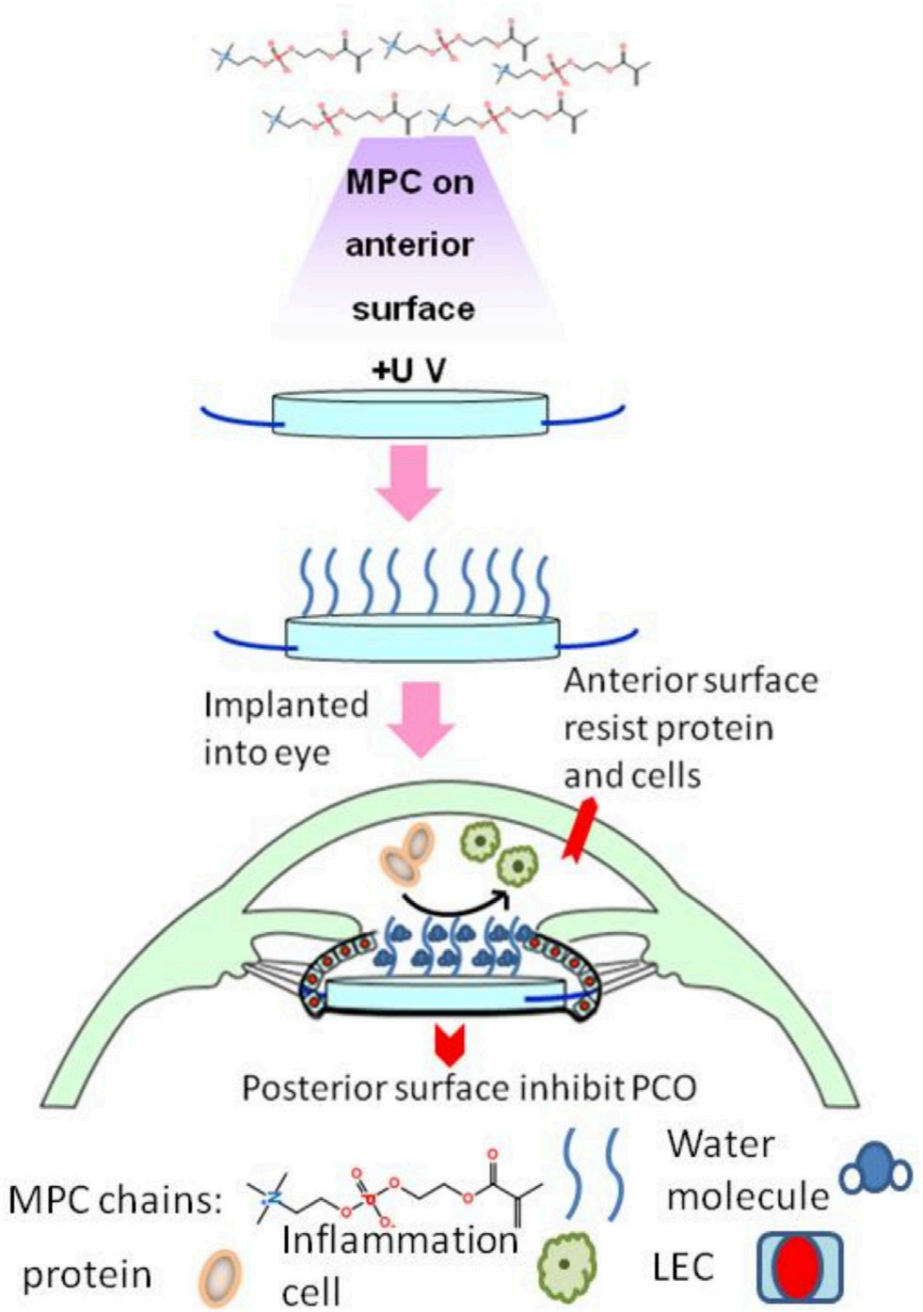
The modified IOL from Huang et al. (2017), with the permission of Elsevier. MPC, 2-methacryloyloxyethyl phosphorylcholine.

The flaw is that plasma technology requires vacuum equipment and takes a long time to accomplish. Huang then tried to graft MPC onto hydrophobic acrylate to construct a hydrophilic interior surface by ultraviolet irradiation (Huang et al., 2017) (Figure 4). This method grafted MPC on the surface of IOL more efficiently. *In vitro* experiments showed that macrophage attachment was inhibited, but LEC migration was found to be increased. This meant that the hydrophilic surface helped reduce anterior inflammatory response but had no promotion of capsular compatibility. From this, they designed IOL in which the front surface links hydrophilic MPCs, while the back surface remains hydrophobic, which was able to control inflammation reaction without causing severe PCO. Han introduced poly MPC brushes to hydrophobic IOL surfaces by surface-initiated reversible addition-fragmentation chain transfer (SI-RAFT) (Han et al., 2017). It allows a wide range of functionalities in the monomers and solvents, including aqueous solutions. Therefore, it is a versatile method of modification. The disadvantages are the complicated preparation process and toxic, colored substances may be involved. Different from previous studies, the incidence of PCO has also been shown *in vivo* to be lower than that with bare IOL.

Other hydrophilic groups, such as PEG and N-vinyl pyrrolidone (NVP), have also been used to modify the IOL surface (Lin et al., 2010; Wang et al., 2013). Few platelets and macrophages were found to adhere postoperatively, proving their good uveal compatibility. And the modified IOL exhibited less corneal edema and exudation in rabbit experiments. In addition, the recombinant hirudin-modified IOL surfaces were reported. The hydrophilicity was mainly provided by -OH. It worked by resisting nonspecific absorption of inflammatory cells and proteins, similar to MPC (Zheng et al., 2016).

#### Materials to Prevent Posterior Capsule Opacification

PCO is caused by the migration and growth of LECs on the posterior capsule and is a major complication after cataract surgery. The incidence of PCO is an important manifestation of capsular compatibility (Awasthi et al., 2009). It has been a hot spot for IOL materials in recent years. Plenty of innovative IOL material modifications provides new ideas for PCO prevention (Han et al., 2018). They mainly focused on inhibiting LECs growing, migrating, and killing LECs. The articles involved are listed in Table 3.

**TABLE 3:** The studies on materials reducing PCO.

	Surface modification	IOL optic materials	Technique	Article
Drug loading modification	DOX*-CHI*-sodium TPP*/HEP*	Hydrophobic acrylic	LBL* assembly	Han et al. (2019)
	PDA*-DOX-MPC*	Hydrophobic acrylic	Immersion	Liu et al. (2021)
	DOX-PAMAM*/HEP		LBL assembly	Chen et al. (2021)
	PEGMA*-s-peptide-DOX	Hydrophobic acrylic	SI-RAFT	Han et al. (2020a)
	DOX-PEGMA-co-GMA*	Hydrophobic acrylic	Immersion	Xia et al. (2021)
	DOX-exosomes	Hydrophobic acrylic	Electroporation	Zhu et al. (2022)
	MTX*	Hydrophobic acrylic	Supercritical fluid	Ongkasin et al. (2020)
	MTX-PLGA*	Hydrophobic acrylic hydrophilic acrylic	Spray coating	Kassumeh et al. (2018)
	CsA*-PLGA		Spin coating	Lu et al. (2022)
	Y27632-PLGA	Hydrophobic acrylic		Lin et al. (2019)
	PEI*-(anti-T/PLL*)4-(anti-T)	Hydrophobic acrylic	LBL assembly	Sun et al. (2014)
	PLGA-bromfenac	Hhydrophobic acrylic	Ultrasonic spray	Zhang et al. (2022)
	HA*-Pac*/CHI	Silicon	LBL assembly	Huang et al. (2021)
	5-Fu-CHI	PMMA	Immersion	Huang et al. (2013b)
	5-Fu-PPGC*	Hydrophobic acrylic	Immersion	Xia et al. (2022)
	GOD*-HRP*-MSNs*	Silicon	Immersion	Huang et al. (2022)
Photodynamic modification	ICG*-PLGA	Immersion		Zhang et al. (2016)
	α-CD-Ce6*-PPEGMA* brush	Hydrophobic acrylic	Supramolecular interaction	Tang et al. (2021)
	Carboxylated CuInS/ZnS quantum dots		Facial activation-immersion	Mao et al. (2020)
	PDA/PEI-PMMA	Hydrophobic acrylic	Immersion	Xu et al. (2021)
	PDA	Hydrophobic acrylic	Immersion	Qie et al. (2022)
Hydrophobic modification	RGD	Hydrophilic acrylic	Plasma	Huang et al. (2014)
Hydrophilic modification	PSBMA* brush coating		SI-RAFT	Wang et al. (2021c)
	PEG* brush		SI-RAFT	Lin et al. (2010)
	HA-CHI	Silicon	LBL assembly	Lin et al. (2015)
	PEG	Hydrogel	Grafting	Bozukova et al. (2007)
	PEG	Hydrophobic acrylic	Plasma	Xu et al. (2016)
	EGPEMA*-co-EA*	Hydrophobic acrylic		Liu et al. (2021)

*DOX, doxorubicin; CHI, chitosan; TPP, tripolyphosphate; HEP, heparin; PDA, polydopamine; MPC, 2-methacryloyloxyethyl phosphorylcholine; PAMAM, polyaminoamide; PEGMA, poly (ethylene glycol) methacrylate; GMA, glycidyl methacrylate; MTX, methotrexate; PLGA, poly (lactic-co-glycolic) acid; CsA, cyclosporine A; PEI, polyethylenimine; PLL, poly-L-lysine; HA, hyaluronic acid; Pac, paclitaxel; ICG, indocyanine green; PPGC, poly (polyethylene glycol methacrylate-co-glycidyl methacrylate-co-coumarin methacrylate); GOD, glucose oxidase; HRP, horseradish peroxidase; MSNs, mesoporous silica nanoparticles; α-CD-Ce6, chlorin e6 grafted α-cyclodextrin; PPEGMA, poly (poly (ethylene glycol) methacrylate); PSBMA, poly (sulfobetaine methacrylate); PEG, poly (Ethylene Glycol); LBL, layer-by-layer; EGPEMA, ethylene glycol phenyl ether methacrylate; EA, 2-(2-ethoxyethoxy) ethyl acrylate.

##### Drug-Loading Modification

Many drugs are used to stop PCO from happening. The traditional method of soaking IOL in the solution can only carry limited drugs and is quickly eluted and metabolized after implantation in the eye (Liu et al., 2013). In this way, the drug works for a short period and cannot meet the long-term effect demands. Therefore, combining drugs with chemical materials on the IOL surface to increase the drug loading and reduce the release rate has been widely studied.

Cell apoptotic drug doxorubicin (DOX) is most commonly carried in drug-loaded materials. Attaching DOX to the IOL surface was thought to influence the growth of LECs. Han et al. (2019) developed an anti-proliferative drug-loaded coating doxorubicin-chitosan-tripolyphosphate (DOX-CHI-TPP, CTDNP). CTDNP nanoparticles were used to make polyelectrolyte multilayer (HEP/CTDNP) n together with heparin by LBL technique. LBL is a solution processing technique for generating multilayer films and coatings with nanoscale thickness control of the overall films and of the hierarchical material composition. Its reaction conditions are mild and its preparation is simple. But it takes a long time and the stability of modification is relatively poor. And drug release was still detected one week after implantation. The migration experiments of LECs *in vitro* demonstrated that the modified IOL had an inhibitory impact on cell proliferation and migration, and the effect was positively related to the number of layers. Another method of loading DOX drugs was proposed by Liu et al. (2021) in 2021. First, a polydopamine (PDA) coating was formed on the surface of the IOL through the self-polymerization of PDA, and the drug loading of DOX on this coating was significantly improved. The hydrophilic MPC was then grafted onto the surface of the drug-loaded coating. The PDA (DOX)-MPC multifunctional coating had good hydrophilicity released drugs slowly and long-term lasting over 3 weeks. It had been proved to suppress the proliferation of LEC by *in vivo* and *in vitro* experiments.

Modified materials not only serve as tools for carrying drugs but may also have additional therapeutic effects. Qin et al. (2021) used cationic dendrimer to improve the therapeutic effect of DOX. DOX-encapsulated polyaminoamide (PAMAM) and heparin were assembled to the IOL surface by the LBL technique. The material itself enhanced the medical effect. Animal experiments found that this modification showed a better prevention effect of PCO than free DOX. This might be due to the ability of cationic dendrimer to enhance cell penetration and autophagy. Han Y. et al. (2020) carried DOX with poly (ethylene glycol) methacrylate (PEGMA) and grafted it to the IOL surface *via* MMP-2-sensitive peptide linkage. In addition to the pharmacological effects of DOX, this modification increased sensitivity to metalloproteinases (MMPs), a protein found to be up-regulated during LEC proliferation. The hydrophilicity of PEGMA itself was also applied by Xia et al. (2021) to work with DOX to further inhibit PCO. Zhu et al. (2022) used exosomes extracted from LECs to carry DOX. They found that the drug was absorbed more effective by the cells and thought this might be due to the targeting ability of the exosomes.

Ongkasin et al. (2020) modified anti-metabolic drug methotrexate (MTX) onto the IOL surface *via* supercritical impregnations. During this process, supercritical CO_2_ was used to dissolve and carry the drug, and ethanol was added to increase drug dissolution. By adjusting the conditions, 8 Mpa, 4 h impregnation achieved 80 days drug-release. Experiments in an *in vitro* model showed that it might contribute to the prevention of PCO in the clinic by inhibiting epithelial-mesenchymal transformation. Kassumeh et al. (2018) sprayed the solution containing MTX, poly (lactic-co-glycolic) acid (PLGA), and isopropanol on the IOL surface to obtain a drug-loaded coating. The drug-loaded coating material, compared to the control group without the drugs, significantly suppressed cell growth and migration. Interestingly, the authors found that modified hydrophilic IOL released more drugs than hydrophobic IOL.

There were other drugs used to manifest PCO, carried by PLGA, which was proved to be a safe and feasible drug carrier (Loureiro and Pereira, 2020). PLGA can effectively protect drugs from degradation and unrestrained release. Solutions of PLGA and cyclosporine A (CsA) were used to modify the IOL surface by Lu et al. (2022). The spin coating method was applied to construct the centrifuging concentric circles drug-loaded coating on the IOL surface. Rotation speed and spin time were adjusted to determine the most appropriate drug release profile. The immunosuppressive drug CsA effectively inhibited the growth of LECs *in vitro* investigations and was thought to control cell apoptosis through autophagic effects. Animal experiments also convinced it. Lin et al. (2019) proposed a PLGA carried, ROCK pathway inhibitor Y27632, to suppress LEC growth. They demonstrated the mechanism by which the ROCK pathway promoted LEC proliferation. And they confirmed that LEC proliferation was reduced when the ROCK pathway was inhibited. Subsequently, modified IOL carrying ROCK pathway inhibitors were also shown to significantly inhibit PCO *in vivo* experiments.

TGF-β is an important factor in promoting lens epithelial-mesenchymal transformation (EMT), which is one of the steps in the occurrence of PCO. It exists in the fluid tissue of the eye and promotes the development of PCO. Sun et al. (2014) firstly applied APGD to create a negatively charged surface on hydrophobic IOL. APGD is an environmentally friendly and energy efficient technique, which has relatively little impact on the bulk material. Then, polyethyleneimine was deposited on the surface. Thirdly, anti-TGF-β2 (anti-T) antibody and poly-L-lysine were placed on the modified surface using the LBL technique for four cycles. The structure could be maintained for three months without difference in optical and physical properties. *In vitro* experiments demonstrated that LEC migration and EMT were greatly suppressed, but proliferation and adhesion were not inhibited significantly. The inhibitory effect of bromfenac on TGF-β was also applied to the modified IOL surface. PLGA-modified extended-release bromfenac was proved effective in preventing PCO (Zhang et al., 2022).

The anti-proliferative drug paclitaxel (Pac) is also thought to possibly inhibit the occurrence of PCO. Huang et al. (2021) made the multilayer coating of hyaluronic acid (HA), CHI, and Pac through the LBL technique. The coating was proven to have good sustained release and biocompatibility. LEC proliferation on the surface of the material was also found to be significantly reduced. 5-fluorouracil chitosan nanoparticles (5-Fu-CSNP) were introduced to effectively suppress PCO (Huang X. et al., 2013). The drug could be continuously released for four days *in vitro* and effectively promoted apoptosis. Subsequently, a light-controlled drug-releasing coating was used to carry 5-Fu (Xia et al., 2022). This modification finely controlled drug release through illumination based on the photo reactivity of coumarin.

In addition to conventional drugs, special chemical reactions were also considered to kill LECs. Horseradish peroxidase (HRP) and glucose oxidase (GOD) were used to catalyse the production of reactive oxygen species, resulting in cell apoptosis. Huang et al. (2022) immobilized GOD and HRP on IOL surface *via* mesoporous silica nanoparticles (MSNs) and obtained positive results.

##### Photodynamic Coating Modification

Using photodynamic therapy-effect coatings instead of carrying drugs may further simplify the production techniques. Due to its exogenous excitation, the effect is more controllable (Han et al., 2016). In 2015, Zhang et al. (2016) had tried laser-activated indocyanine green (ICG) to inhibit LEC proliferation and migration. And PLGA was used to cover the ICG coating to prolong the residence time of the drug. *In vivo* pharmacodynamic experiments demonstrated that the ICG-IOL could successfully inhibit PCO. Moreover, both the ICG and PLGA were easily metabolized, indicating little harm to the human body.

Another photodynamic coating was presented to control PCO (Tang et al., 2021). The poly [poly (ethylene glycol) methacrylate] (PPEGMA) brush was firstly established on the surface of IOL by SI-RAFT. The chlorin e6 grafted α-cyclodextrin (α-CD-Ce6) then was attached to the PPEGMA brush as a photosensitizer. When exposed to light, α-CD-Ce6 induced apoptosis by producing reactive oxygen species (ROS). Illumination has been shown to not influence the optical properties and biocompatibility of the material. And the effectiveness was also proved in animal experiments. CuInS/ZnS quantum dots (ZCIS QDs) was synthesized and carboxylated by Mao et al. (2021). And it was modified onto the non-optical surface of IOL by the facial activation-immersion method. ZCIS QDs inhibited LEC growth by releasing localized heat under mild near-infrared light irradiation. But its biocompatibility was not known due to the lack of *in vivo* experiments.

Xu et al. (2021) introduced a photothermal IOL (PT-IOL) with a mussel-inspired coating. Dopamine hydrochloride was dissolved with CuSO_4_ 5H_2_O and H_2_O_2_ (30%) for PDA deposition. Hydrophobic acrylic IOL were immersed respectively in PDA and polyethyleneimine (PEI) solutions to form the coating on the non-optical surface. It was found that the thickness of the coating was positively related to the soaking time, and 40 min immersion obtained the most appropriate coating. The irradiation of NIR was converted to thermal energy through this material, and elevated temperature around the PT-IOL proved to be effective in killing LECs. Biological experiments, as well, proved the preventive effect on PCO. Another PDA surface modification was introduced by Qie and proved to be effective in blocking PCO Qie et al. (2022). Its mechanism was thought to induce apoptosis through reactive oxygen species.

##### Hydrophobic Modification

Hydrophilic materials are generally considered to be more likely to cause severe PCO than hydrophobic materials. The sticky surface of the hydrophobic material largely prevents the migration of LECs. LECs rapidly fibrosis, degenerate, and even die around the hydrophobic edges, which do not cover the center of IOL. However, hydrophilic materials are more suitable for LECs growth. It will block the optical part, seriously affecting the vision (Bertrand et al., 2014). A cell adhesion molecule (RGD peptide) that can be recognized by a variety of integrins to adhere to many cells was used to compensate for the defects of hydrophilic materials (Huang et al., 2014). Oxygen plasma was involved in the surface modification process. The modified material is highly hydrophilic but exhibits significant LEC adhesion. It meant that RGD might play a role in inhibiting PCO.

##### Hydrophilic Modification

On the contrary, Wang R. et al. (2021) tried to modify the surface of hydrophobic IOL with hydrophilic polymer poly (sulfobetaine methacrylate) (PSBMA); some of researches (Bozukova et al., 2007; Xu et al., 2016; Lin Q. et al., 2017) modified IOL surface with PEG. PSBMA and PEG brushes were coated on to IOL surface by the SI-RAFT technique. Cellular experiments showed that initial LEC adhesion to IOL surfaces was reduced. Recently, bulk modification was used to graft EGPEMA-co-2-(2-ethoxyethoxy) ethyl acrylate (EA) polymer onto IOL surfaces. And it was believed to be easier and more efficient to produce compared to other surface modifications. However, previous studies had suggested that hydrophilic materials were more suitable for cell growth due to their high water content. This seems to contradict their results. Thus, further research is expected to verify the specific impact of hydrophilic materials on the development of PCO.

### Antibacterial Surface Modification

Postoperative endophthalmitis is caused by bacteria, of which more than 95% are Gram-positive, entering the eye with the instrument or IOL during surgery (Garg et al., 2017). The probability of postoperative endophthalmitis is very low. But once endophthalmitis occurs, it can be serious and even lead to blindness (Durand, 2017; Fan et al., 2021). To prevent endophthalmitis, the surgeons use antibiotic eye drops (Friling et al., 2013). Drug-carrying IOL have also been of concern. Some studies carried multiple drugs, giving IOLs the effect of preventing multiple complications (Topete et al., 2020). This requires the innovation of IOL materials to meet the needs of carrying antibiotics and sustained release. The articles involved are listed in Table 4.

**TABLE 4:** The studies on materials reducing endophthalmitis.

Drug	Surface modification	IOL optic materials	Technique	Article
MXF*	AMPS*/SBMA*	Hydrophilic acrylic	Plasma grafting	Pimenta et al. (2017)
	PHEMA coating	Hydrophilic acrylic	Argon plasma-assisted grafting	Vieira et al. (2017)
AMP*	PSS*-PEI	PMMA	LBL assembly	Manju and Kunnatheeri, (2010)
Vancomycin	Poly 2/polyanion/vancomycin/polyanion		LBL assembly	Shukla et al. (2011)
NFX*	Octadecyl isocyanate	Hydrophilic acrylic	Grafting	Anderson et al. (2009)
Gentamycin	PPPE* IOL-PDA	Hydrophobic acrylic		Yang et al. (2021)
No antibiotics	p (DMAEMA*-CO-MPC) brush	Silicon	SI-RAFT	Wang et al. (2017)
	p (VBC-CO-DMAEMA)		Chemical vapor deposition	Choi et al. (2020)
	HA*-CHI*	Silicon	Polyelectrolyte deposition	Lin et al. (2017a)

*MXF, moxifloxacin; AMP, ampicillin; NFX, norfloxacin; AMPS, 2-acrylamido-2-methylpropane sulfonic acid; SBMA, (2-(methacryloyloxy)ethyl) dimethyl-(3-sulfopropyl) ammonium hydroxide; PSS, poly (sodium 4-styrenesulfonate); PPPE, poly (2-phenoxyethyl methacrylate-co-2-phenoxyethyl acrylate-co-2-ethylhexyl methacrylate); DMAEMA, 2-(dimethylamino)-ethyl methacrylate; HA, hyaluronic acid; CHI, chitosan polyelectrolyte.

Moxifloxacin (MXF), a type of broad-spectrum antibacterial fluoroquinolone, is often used to prevent endophthalmitis after cataract surgery. Pimenta et al. (2017) grafted [2-(methacryloyloxy)ethyl] dimethyl-(3-sulfopropyl) ammonium hydroxide (SBMA) or 2-acrylamido-2-methylpropane sulfonic acid (AMPS) onto the surface of IOL through argon plasma-assisted copolymerization. AMPS showed higher MXF carrying capacity and longer drug release time with 21 days. The modified drug-loaded materials were stored 30 days after sterilization. Released MXF remained effective against *S. aureus* and Staphylococcus epidermidis after 12 days, consistent with the recommended duration of antibiotic therapy after surgery. Vieira et al. (2017) used poly HEMA modified IOL to load MXF. Argon exposure for 3 min appeared higher hydrophilicity. Controlled experiments compared the status of drug loading under different conditions, 15 h at 37°C, with 100 rpm proved to be the best loading condition.

Adjusting the bulk material can further extend the duration of maintenance. Topete et al. (2021a) added functional monomers in hydrogels to regulate the release of MXF. They tried acrylic acid (AA), methacrylic acid (MAA), 4-vinylpiridine (4-VP), and MAA+4-VP, as well as molecular imprinting techniques. The addition of MMA functional monomers alone was considered the best way to carry MXF. The physical properties and transparency of the modified IOL material were not affected. Antimicrobial tests showed that released MFX remained active against *S. aureus*.

Other types of antibiotics were applied to prevent endophthalmitis. Manju and Kunnatheeri (2010) created a multilayer coating IOL to load ampicillin (AMP). LBL technique was applied. IOL was sunk alternatively in a solution of PEI and another of poly (sodium 4-styrenesulfonate) (PSS) after being immersed in an ammonia solution. The drug release was observed to reach 91% of the total after 4 days, with a plateau in approximately 5 days. Shukla et al. (2011) used the same technique to carry vancomycin. Likewise, the modified IOL material exhibits good drug loading capacity. Octadecyl isocyanate worked as a hydrophobic barrier to limit drug release, exhibiting a sustained-release effect (Anderson et al., 2009). Norfloxacin (NFX) was dissolved in the solution to modify PHEMA hydrogels, and a significant bacteriostatic effect was demonstrated. Yang (Xiang et al., 2021) grafted PDA and gentamicin on the hydrophobic acrylate IOL synthesized of poly (2-phenoxyethyl methacrylate-co-2-phenoxyethyl acrylate-co-2-ethylhexyl methacrylate) (PPPE). It was proven to be effective against bacteria and reduce PCO. For different types of antibacterial drugs, there are the most suitable modification conditions, respectively. Therefore, it is necessary to develop more drug-loaded modifications.

**FIGURE 5:**
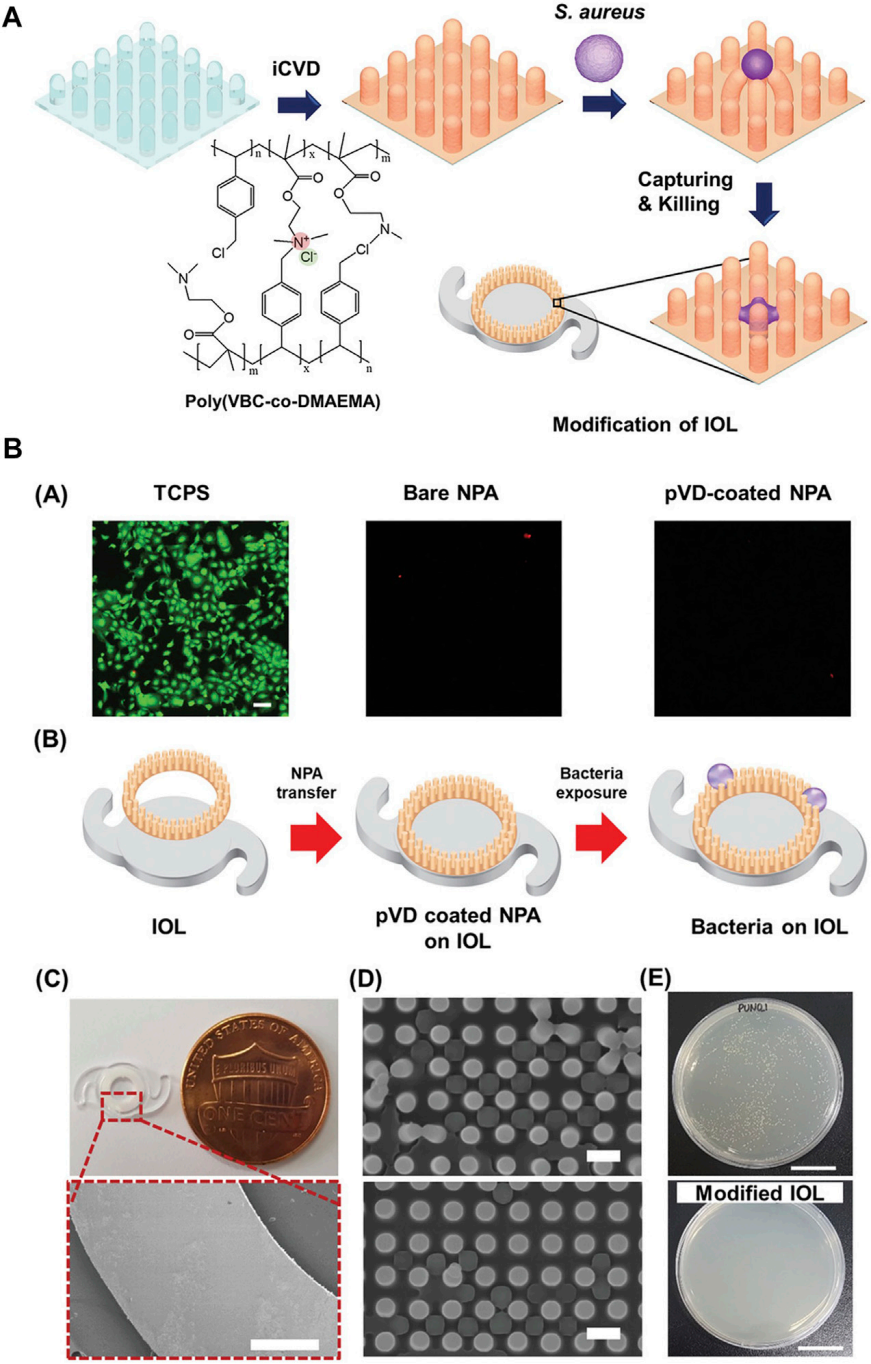
**(A)** Schematic of the ionic polymer-coated NPA. **(B)**. **(A)** Live/dead staining and adhesion test of corneal endothelial cells on each NPA after a 1 day culture. There were no usable and adhered cells on the NPA due to the dimension of the NPA structure. The scale bar is 100 µm. **(B–E)** Modification of IOL with pVD-coated NPA for the antibacterial property. **(B)** Schematic of the monolithic integration strategy of pVD-coated NPA onto the IOL. **(C)** Photographic image of the modified IOL and SEM images of the modified IOL edge. Scale bars are 500 µm. **(D)** SEM images of the NPA after exposure to *S. aureus*. Scale bars is 1 µm. **(E)** Representative images after colony-counting assay with control and modified IOL. The scale bars are 1 cm. Reprinted from Choi et al. (2020), with the permission of Wiley. NPA, polymeric nanopillar array; pVD, crosslinked ionic polymer thin film.

The modified materials can also exert antibacterial effects. Wang et al. (2017) grafted 2-(dimethylamino)-ethyl methacrylate-co-2-methacryloyloxyethyl phosphorylcholine p (DMAEMA-co-MPC) brushes on PDMS, which was one of the commonly used IOL materials. SI-RAFT was involved in this procedure. 1-bromoheptane (25 v/v%) was applied to quaternize the polymer, and meanwhile, invest the antibacterial function. Shake-flask culture and live/dead staining confirmed the suppression of *S. aureus*. Furthermore, p (DMAEMA-co-MPC) increased surface hydrophilicity. The modified IOL was proved to be able to reduce bacterial adhesion and biofilm formation on the surface. Likewise, a polymeric nanopillar array surface modification was engineered to disrupt bacterial membranes (Choi et al., 2020). 4-vinylbenzyl chloride (VBC) and DMAEMA were deposited onto the IOL surface to form p (VBC-co-DMAEMA) modification. In addition to being antibacterial, it has also been shown to be somewhat resistant to PCO (Figure 5). HA/CHI modification have also been proved to inhibit bacterial growth (Lin Q. K. et al., 2017). This was attributed to the ability of reducing bacterial adhesion. And similar to LECs, HA/CHI modification inhibit the cells to grow on the surface of IOL, thus preventing the PCO (Lin et al., 2015).

### Surface Modification as Sensors

**FIGURE 6:**
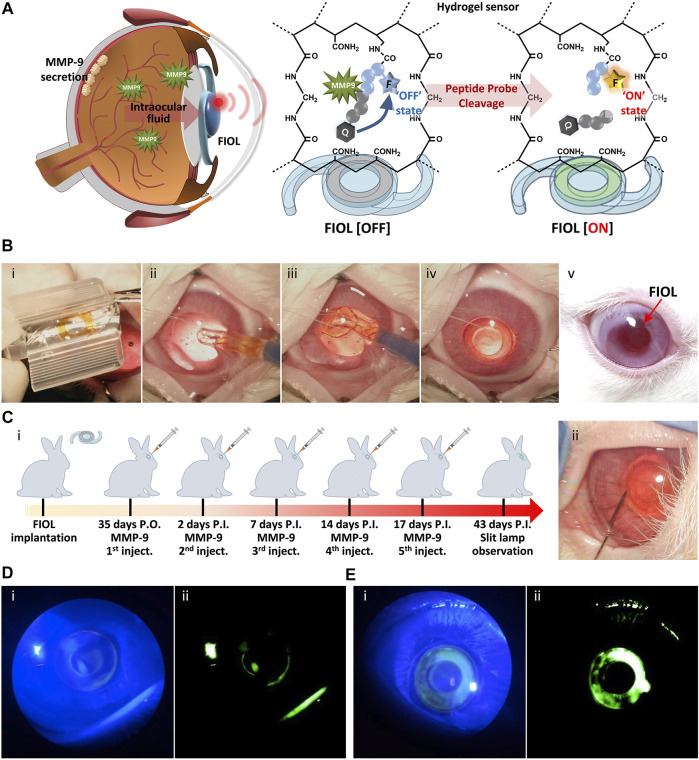
*In vivo* monitoring of MMP-9 using FIOL. **(A)** Schematic illustration of the reaction mechanism of FIOL implanted inside the eye. **(B)** Representative photographs of the process of FIOL implantation during *in vivo* rabbit cataract surgery: **(i)** Loading of FIOL into the cartridge of the injector, **(ii,iii)** Insertion of FIOL into the posterior chamber of the eye and **(iv)** final implantation status of FIOL in the eye. **(v)** FIOL implanted in the eye maintained proper position without any adverse response, including immune reactions, over 7 weeks postoperatively. **(C) (i)** Schematic illustration of *in vivo* testing for MMP-9 sensing of FIOL inside the eye and **(ii)** representative photograph of intraocular MMP-9 injection after FIOL implantation. Slit-lamp photographs of FIOL inside the eye after **(D)** 2 days P.I. and **(E)** 43 days P.I., with **(i)** cobalt blue filtered light and **(ii)** green filtered images, respectively. Reprinted from Shin et al. (2020), with the permission of Elsevier.

In recent years, attempts have been made to impose additional uses for IOL. The development of biosensing technology makes it possible for IOL to be used as a sensing device to detect special biomolecules or the environment in the eye (Yang et al., 2021). And some biosensing devices also involve material development. Shin et al. (2020) developed a fluorescent IOL (FIOL) based on diacrylamide-group-modified PEG diacrylamide (PEGDAAm) hydrogels. Specific peptide probes were attached to IOL to detect the concentration of MMP-9 in aqueous humor, which is a biomarker reflecting neurological diseases (Figure 6). Other sensors for monitoring intraocular pressure (Narasimhan et al., 2018) and glucose concentration (Yang et al., 2018) are also being developed, but they are not well integrated with intraocular lens materials. We expect more research in related fields in the future.

## Conclusion

The commonly used materials for commercialized IOL today are silicon, hydrophilic and hydrophobic acrylates. Liquid and shape-memory materials have been developed to insert through smaller surgical incisions, and even if an intact capsular bag can be maintained, the ability to accommodate is preserved. Light-reactive molecules are used to make IOL that can adjust the refractive power. Usually, surface modification of polymer materials is considered to improve biocompatibility, involving LPL, plasma, grafting, coating, and other methods. A variety of molecules are modified to the surface of the IOL material to complement specific effects by changing the properties of the surface. Carrying drugs on the surface of the IOL prevents postoperative complications, such as intraocular infections and PCO, which is also an indicator of capsular biocompatibility.
